# High stress, lack of sleep, low school performance, and suicide attempts are associated with high energy drink intake in adolescents

**DOI:** 10.1371/journal.pone.0187759

**Published:** 2017-11-14

**Authors:** So Young Kim, Songyong Sim, Hyo Geun Choi

**Affiliations:** 1 Department of Otorhinolaryngology-Head & Neck Surgery, CHA Bundang Medical Center, CHA University, Seongnam, Korea; 2 Department of Statistics, Hallym University, Chuncheon, Korea; 3 Department of Otorhinolaryngology-Head & Neck Surgery, Hallym University College of Medicine, Anyang, Korea; McMaster University, CANADA

## Abstract

**Objective:**

Although an association between energy drinks and suicide has been suggested, few prior studies have considered the role of emotional factors including stress, sleep, and school performance in adolescents. This study aimed to evaluate the association of energy drinks with suicide, independent of possible confounders including stress, sleep, and school performance.

**Methods:**

In total, 121,106 adolescents with 13–18 years olds from the 2014 and 2015 Korea Youth Risk Behavior Web-based Survey were surveyed for age, sex, region of residence, economic level, paternal and maternal education level, sleep time, stress level, school performance, frequency of energy drink intake, and suicide attempts. Subjective stress levels were classified into severe, moderate, mild, a little, and no stress. Sleep time was divided into 6 groups: < 6 h; 6 ≤ h < 7; 7 ≤ h < 8; 8 ≤ h < 9; and ≥ 9 h. School performance was classified into 5 levels: A (highest), B (middle, high), C (middle), D (middle, low), and E (lowest). Frequency of energy drink consumption was divided into 3 groups: ≥ 3, 1–2, and 0 times a week. The associations of sleep time, stress level, and school performance with suicide attempts and the frequency of energy drink intake were analyzed using multiple and ordinal logistic regression analysis, respectively, with complex sampling. The relationship between frequency of energy drink intake and suicide attempts was analyzed using multiple logistic regression analysis with complex sampling.

**Results:**

Higher stress levels, lack of sleep, and low school performance were significantly associated with suicide attempts (each P < 0.001). These variables of high stress level, abnormal sleep time, and low school performance were also proportionally related with higher energy drink intake (P < 0.001). Frequent energy drink intake was significantly associated with suicide attempts in multiple logistic regression analyses (AOR for frequency of energy intake ≥ 3 times a week = 3.03, 95% CI = 2.64–3.49, P < 0.001).

**Conclusion:**

Severe stress, inadequate sleep, and low school performance were related with more energy drink intake and suicide attempts in Korean adolescents. Frequent energy drink intake was positively related with suicide attempts, even after adjusting for stress, sleep time, and school performance.

## Introduction

Energy drinks are soft drinks with a high caffeine content, with some drinks containing as much as 505 mg of caffeine per serving and other substances including high dose of sugar, some vitamins, including riboflavin, pyridoxine, nicotinamide, other B vitamins, and various herbal derivatives, which could act as stimulants [1, 2]. Energy drinks have been increasingly consumed, especially among adolescents, to sustain alertness and boost energy [3]. In addition, the considerable adolescents consume energy drink to just enjoy its taste or celebrate special occasion with alcohol [4]. In Canada, a previous study indicated that 73.6% of the young population had regularly consumed energy drinks [5], and approximately 30–50% of the young population worldwide uses energy drinks [6]. However, the effects of these drinks on reducing fatigue and boosting energy are controversial and short-lasting [1]. Several studies have reported fatal effects of caffeine abuse including suicide, with some conflicting findings [7]; suicide attempts using high caffeine intoxication have been reported with blood caffeine concentrations reaching as high as 170 mg/l [8]. The main active compound of caffeine is one of the most popular substances worldwide, which has occasional potential to abuse [9].

Youth are more susceptible to the adverse health effects of caffeine due to their lower tolerance of the substance, smaller body surface area for drug distribution, and less effective metabolic excretion capabilities. In addition, adolescents are vulnerable to emotional changes, a heavy burden of school work, and behavioral problems. Several previous studies have reported adverse health outcomes related to energy drink use in adolescents [10, 11]. Specifically, energy drink consumption has been associated with mood and behavioral disorders in youth [6].

Globally, suicide accounts for approximately 12–14.2 per 100,000 of 15–24 years olds [12]. In Korea, the mortality rate of suicide has been increasing in the general population and in adolescents by approximately 28.5 and 7.8–9.7 per 100,000, respectively [13, 14].

The present study aimed to investigate the association between energy drink intake and suicide in a large Korean adolescent population. The running hypothesis of this study was that energy drink intake might elevate suicide. This study assumed that school performance was linked with both energy drink consumption and suicide, as Korean adolescents experience a high academic burden due to the competitive nature of university entrance examinations.

## Materials and methods

### Study population and data collection

The Institutional Review Board of the Centers for Disease Control and Prevention of Korea (KCDC) approved this study (2014-06EXP-02-P-A). Written informed consent was obtained from each participant prior to the survey. As this web-based survey was performed at school with a large number of participants, informed consent from their parents was exempted. This consent procedure was approved by the KCDC IRB.

This cross-sectional study used data from the Korea Youth Risk Behavior Web-based Survey (KYRBWS). The KYRBWS covers the nation using statistical methods based on designed sampling and adjusted weighted values; data from the 2014 and 2015 KYRBWS were analyzed. These data were collected by the KCDC. Korean adolescents from 7^th^ through 12^th^ grade completed the self-administered questionnaire voluntarily and anonymously. The validity and reliability of the KYRBWS have been documented by other studies [15, 16]. Based on 43 regions (considering administrative district, geographic accessibility, the number of schools, and the population size) and schools, the initial population was stratified into 129 levels for sample distribution. The sample was selected using stratified, two-stage (schools and classes) clustered sampling based on data from the Education Ministry. Sampling was weighted by statisticians, who performed post-stratification analyses and considered the non-response rates and extreme values. Further details regarding the methods have been described on the KYRBWS website [17].

**Fig 1:**
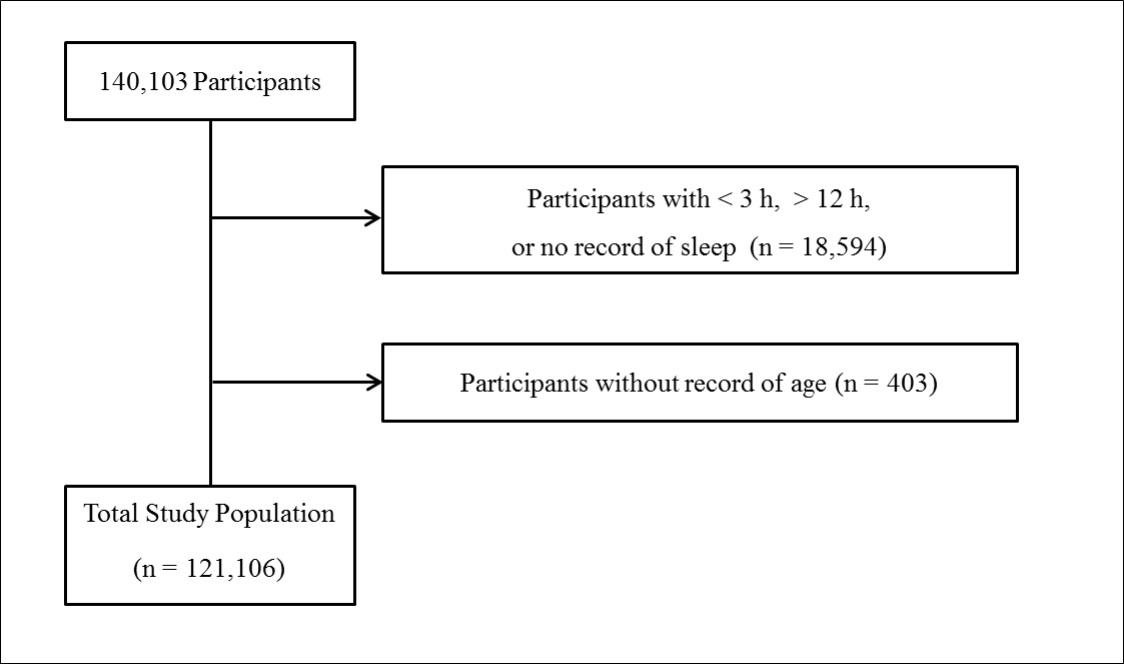
A schematic illustration of participant selection in the present study.

The response rates of the KYRBWS were 97.2% (72,060/74,167) and 96.7% (68,043/70,362) in 2014 and 2015, respectively. Of the 140,103 total participants, we excluded the following participants from this study: participants who slept less than 3 hours or more than 12 hours or had no record of sleep duration (18,594 participants) and participants who did not provide their age (403 participants). Finally, 121,106 participants (60,696 male and 60,410 female) 12 through 18 years old were included in this study (Fig 1).

### Survey

Region of residence was divided into 3 groups by administrative district: large city, small city, and rural area. Self-reported economic level was measured at 5 levels from highest to lowest. Parents’ education level was divided into 4 groups: graduated college or higher, graduated high school, graduated middle school or lower, and unknown or no parent. Participants who did not know their parents’ educational level or who did not have a parent were not excluded, as their exclusion could increase the missing values in participants with a relatively lower economic level.

Participants’ stress level was divided into 5 groups: severe, moderate, mild, a little, and no stress. Participants classified their stress level into 5 categories: I feel a lot of stress (severe), I feel some stress (moderate), I feel a little stress (mild), I rarely feel stress (a little), and I do not feel stress (no stress). Duration of sleep in the last 7 days was also evaluated. The participants’ sleeping and waking time were measured to within 10 min and were subtracted from each other to obtain the duration of sleep. Mean daily sleep time was calculated by adding their weekday and weekend sleep times with 5/7 and 2/7 weights, respectively. Sleep time was divided into 6 groups: < 6 h; 6 ≤ h < 7; 7 ≤ h < 8; 8 ≤ h < 9; and ≥ 9 h. Participants were also asked about their performance at school in the past 12 months, and their grades were divided into 5 levels: A (highest), B (middle, high), C (middle), D (middle, low), and E (lowest).

Participants were asked about their frequency of consuming energy drinks (RedBull, Hot6, or Bacchus) in the past 7 days, and their responses were categorized into 7 groups: ≥ 3 times a day; 2 times a day; once a day; 5–6 times a week; 3–4 times a week; 1–2 times a week; and 0 times a week. Because of the low frequency of participants in the high consumption group, frequency of energy drink consumption was regrouped into 3 groups: ≥ 3 times a week; 1–2 times a week; and 0 times a week. Suicide attempts in the past 12 months were also assessed using a questionnaire: Have you ever experienced a trial to suicide in recent 12 months? Participants who had attempted suicide in the past 12 months were recorded as positive.

### Statistical analysis

This study extended previous investigations by using stratified analyses. Previous studies did not adjust confounders such as sleep time and school performance. Suicide attempts can be affected by a number of emotional problems that influence each other and are intertwined. Stress is one of the most commonly associated and prevalent risk factors of suicide. For adolescents, sleep deprivation and academic load are important risk factors for stress and suicide. Thus, stress, sleep time, and school performance should be considered when evaluating the relationship between energy drink consumption and suicide. The confounders of stress, sleep time, and school performance were adjusted in this study. Energy drink intake was first analyzed regarding its relations with sleep, stress, and school performance, all of which can affect suicide. Then, the association between energy drink intake and suicide was analyzed, adjusting for sleep, stress, and school performance. Differences in general characteristics according to intake of highly caffeinated drinks were calculated using linear regression analysis with complex sampling for age. The rate differences in sex, region of residence, economic level, parental education level, sleep time, stress level, and school performance were compared using chi-square tests with Rao-Scott correction.

The odds ratios (ORs) of suicide attempts in terms of sleep time, stress level, and school performance were calculated using multiple logistic regression analysis with complex sampling adjusting for age, sex, region of residence, economic level, and parents’ education level.

The ORs of energy drink consumption by sleep time, stress level, and school performance were calculated using the following model: ordinal logistic regression analysis with complex sampling adjusting for age, sex, region of residence, economic level, parents’ educational level, sleep time, stress level, and school performance. The ORs of energy drink consumption represented the relative risk for a 1-group increase in energy drink use (e.g. from 1–2 timmes a week to ≥ 3 times a week). Multinomial logistic regression analysis with complex sampling was also performed, adjusting for age, sex, region of residence, economic level, parents’ education level, sleep time, stress level, and school performance.

Finally, the ORs of energy drink use by suicide attempt were calculated using multiple logistic regression analysis with complex sampling adjusted for age, sex, region of residence, economic level, parents’ education level, sleep time, stress level, and school performance.

Two-tailed analyses were conducted, and *P*-values lower than 0.05 were considered to indicate significance. Adjusted ORs (AORs) and 95% confidence intervals (CIs) were calculated. All results are presented as weighted values, based on the weights recommended by the KYRBWS. The statistical analyses were conducted using SPSS ver. 21.0 (IBM, Armonk, NY, USA).

## Results

In total, 88.6% (107,248/121,106), 8.5% (10,327/121,106), and 2.9% (3,531/121,106) of the participants consumed energy drinks 0 times, 1–2 times, and 3 or more times per week, respectively (Table 1). Additionally, 2.4% (2,867/121,106) of the participants had attempted suicide. The frequency of energy drink consumption differed between participants who had and had not attempted suicide. Among participants with suicide attempts, 77.5% (2,222/2,869), 12.6% (360/2,869), and 9.9% (285/2,869) had consumed energy drinks 0 times, 1–2 times, and 3 or more times per week, respectively. All other characteristics including age, sex, region of residence, economic level, maternal and paternal educational levels, sleep time, stress level, and school performance differed significantly between energy drink groups (all P < 0.001). Thus, all of these variables were adjusted for in the ordinal logistic regression analysis of the relations between energy drinks consumption and independent variables of stress level, sleep time, self-assessment of school performance, and suicide trials.

**Table 1:** General characteristics of participants according to energy drinks consumption.

Characteristics		High energy drink			P Value
		0/week	1-2/week	≥ 3/week	
Total Number (n, %)		107,248 (88.6)	10,327 (8.5)	3,531 (2.9)	
Mean Age (y)		15.0	15.0	15.2	<0.001*
Sex (n, %)					<0.001†
	Male	52,534 (86.6)	5,947 (9.8)	2,215 (3.6)	
	Female	54,714 (90.6)	4,380 (7.3)	1,316 (2.2)	
Region (n, %)					<0.001†
	Large City	48,023 (88.9)	4,425 (8.2)	1,575 (2.9)	
	Small City	50,785 (88.6)	4,890 (8.5)	1,630 (2.8)	
	Rural Area	8,440 (86.3)	1,012 (10.3)	326 (3.3)	
Economic level (n, %)					<0.001†
	Highest	8,410 (86.2)	906 (9.3)	440 (4.5)	
	Middle High	28,345 (88.9)	2,634 (8.3)	911 (2.9)	
	Middle	52,007 (89.1)	4,836 (8.3)	1,500 (2.6)	
	Middle Low	15,142 (88.5)	1,486 (8.7)	472 (2.8)	
	Lowest	3,344 (83.2)	465 (11.6)	208 (5.2)	
Education, Father (n, %)					<0.001†
	Unknown	20,834 (86.5)	2,451 (10.2)	788 (3.3)	
	Middle School	2,839 (86.5)	318 (9.7)	124 (3.8)	
	High School	31,576 (89.1)	2,928 (8.3)	943 (2.7)	
	College, or over	51,999 (89.2)	4,630 (7.9)	1,676 (2.9)	
Education, Mother (n, %)					<0.001†
	Unknown	19,845 (86.0)	2,418 (10.5)	800 (3.5)	
	Middle School	2,463 (87.3)	270 (9.6)	88 (3.1)	
	High School	40,121 (89.2)	3,662 (8.1)	1,174 (2.6)	
	College, or over	44,819 (89.2)	3,977 (7.9)	1,469 (2.9)	
Sleep time (n, %)					<0.001†
	< 6 h	24,039 (85.7)	2,758 (9.8)	1,252 (4.5)	
	≥ 6 h, < 7 h	27,096 (89.1)	2,468 (8.1)	830 (2.7)	
	≥ 7 h, < 8 h	27,785 (89.8)	2,466 (8.0)	674 (2.2)	
	≥ 8 h, < 9 h	19,021 (89.9)	1,705 (8.1)	442 (2.1)	
	≥ 9 h	9,307 (88.1)	930 (8.8)	333 (3.2)	
Stress level (n, %)					<0.001†
	No	3,605 (89.1)	288 (7.1)	152 (3.8)	
	A little	18,274 (91.0)	1,425 (7.1)	380 (1.9)	
	Mild	47,279 (89.4)	4,354 (8.2)	1,243 (2.4)	
	Moderate	28,953 (87.5)	3,103 (9.4)	1,037 (3.1)	
	Severe	9,137 (83.0)	1,157 (10.5)	719 (6.5)	
Performance at School (n, %)					<0.001†
	A	13,625 (89.9)	1,069 (7.1)	457 (3.0)	
	B	2,7884 (90.3)	2,243 (7.3)	745 (2.4)	
	C	30,356 (89.2)	2,802 (8.2)	885 (2.6)	
	D	24,905 (87.5)	2,706 (9.5)	866 (3.0)	
	E	10,478 (83.4)	1,507 (12.0)	578 (4.6)	
Suicide trial					<0.001†
	Yes	2,222 (77.5)	360 (12.6)	285 (9.9)	
	No	105,026 (97.9)	9,967 (8.4)	3,246 (2.7)	

* Linear regression analysis with complex sampling, Significance at P < 0.05.

† Chi-square test with Rao-Scott correction, Significance at P < 0.05.

Subjective stress level, sleep time, and school performance showed significant associations with energy drink consumption in the ordinal logistic regression analysis (Table 2). The associations of subjective stress level, sleep time, and school performance were consistent in the multinomial logistic regression analyses (S1 Table). In addition, both male and female subgroup analyses showed similarly significant associations of subjective stress level, sleep time, and school performance with suicide attempts (S2 Table).

**Table 2:** Ordinal logistic regression analyses with complex sampling of subjective stress level, sleep time, and self-assessment of school performance for energy drinks consumption.

		AOR (95% CI)	P-value
Subjective stress level			< 0.001*
	No	1	
	A little	0.89 (0.79–0.99)	
	Mild	1.10 (0.98–1.22)	
	Moderate	1.34 (1.20–1.50)	
	Severe	1.90 (1.68–2.14)	
Sleep time			< 0.001*
	< 6 h	1.59 (1.50–1.68)	
	≥ 6 h, < 7 h	1.13 (1.07–1.20)	
	≥ 7 h, < 8 h	1	
	≥ 8 h, < 9 h	0.97 (0.92–1.03)	
	≥ 9 h	1.14 (1.06–1.23)	
Performance at School			< 0.001*
	A	1	
	B	1.01 (0.94–1.08)	
	C	1.14 (1.07–1.22)	
	D	1.30 (1.22–1.39)	
	E	1.61 (1.49–1.74)	

* Significance at P < 0.05.

Because independent variables of stress level, sleep time, and self-assessment of school performance have associations with suicide trials as well as energy drink consumption, the relations between the independent variables and suicide trials were analyzed. Higher stress levels, lack of sleep, and low school performance were positively associated with suicide attempt (AOR for severe stress = 9.87, 95% CI = 7.22–13.50, P < 0.001; AOR for < 6 h of sleep = 2.01, 95% CI = 1.79–2.26, P < 0.001; AOR for school performance in group E = 2.44, 95% CI = 2.09–2.84, P < 0.001, Table 3).

**Table 3:** Multiple logistic regression analyses with complex sampling of subjective stress level, sleep time, and self-assessment of school performance (independent variables) for suicide trial (dependent variable).

		AOR (95% CI)	P-value
Subjective stress level			< 0.001*
	No	1	
	A little	0.46 (0.32–0.67)	
	Mild	1.06 (0.77–1.45)	
	Moderate	3.28 (2.39–4.49)	
	Severe	9.87 (7.22–13.50)	
Sleep time			< 0.001*
	< 6 h	2.01 (1.79–2.26)	
	≥ 6 h, < 7 h	1.29 (1.15–1.44)	
	≥ 7 h, < 8 h	1	
	≥ 8 h, < 9 h	0.80 (0.70–0.92)	
	≥ 9 h	1.14 (0.98–1.33)	
Performance at School			< 0.001*
	A	1	
	B	1.02 (0.88–1.18)	
	C	1.15 (0.99–1.33)	
	D	1.47 (1.27–1.69)	
	E	2.44 (2.09–2.84)	

* Significance at P < 0.05.

Therefore, the stress level, sleep time, and self-assessment of school performance were adjusted to analyze the relation between energy drink consumption and suicide trials. Frequent energy drink consumption was significantly associated with suicide attempts in the multiple logistic regression analyses (Table 4). The AORs of suicide attempts were 1.46 (95% CI = 1.30–1.65) and 3.03 (2.64–3.49) for the groups consuming energy drinks 1–2 times/week and ≥ 3 times/week, respectively (P < 0.001). The subgroup analyses by gender showed comparable results (S3 Table).

**Table 4:** Multiple logistic regression analyses with complex sampling of energy drinks consumption for suicide trial.

		AOR (95% CI)	P-value
High energy drink			< 0.001*
	0/week	1	
	1-2/week	1.46 (1.30–1.65)	
	≥ 3/week	3.03 (2.64–3.49)	

* Significance at P < 0.05.

## Discussion

In the present study, frequent energy drink intake was found to increase the OR of suicide attempts in the Korean adolescent population after adjusting for stress, sleep, and school performance. These major health problems of suicide as well as stress, sleep, and school performance should be concerned in adolescents with frequent energy intake. Considering the increasing consumption of energy drinks in recent years, the preventive strategies are warranted to prevent these frequent energy drink-related adverse health outcomes.

A few prior studies reported results that conflict with those of the present study. These discrepancies could originate from the differences in the amount of caffeine intake between studies. A prospective cohort study suggested a lower risk of suicide with frequent caffeine consumption [18]. However, that study was based on cups of caffeinated coffee, which might contain less caffeine than energy drinks. Furthermore, most prior studies did not sufficiently consider potential confounders of suicide.

The present study adjusted for demographic and socioeconomic factors, stress, sleep, and school performance to evaluate the relationship between energy drink intake and suicide. The stress level, sleep time, and school performance were significantly associated with the frequency of energy drink intake and were also related to suicide attempts. In accordance with our results, several researchers suggested the effects of energy drink consumptions on mood, sleep disturbance, and performance in adolescents [19, 20]. A recent study reported a significant association of energy drink intake with sleep dissatisfaction, stress, depressive mood, and suicide attempts in Korean adolescents [21]. Another previous study with Korean adolescents demonstrated a significant association between higher caffeine intake and lower academic performance, depression, and insomnia [22].

Caffeine stimulates the central nervous and cardiac systems [7]. Within therapeutic ranges, caffeine inhibits adenosine receptors and phosphodiesterase, thereby elevating the intracellular calcium concentration, and this process releases noradrenaline and sensitizes dopamine receptors. However, at intoxicating levels, caffeine causes abdominal pain, vomiting, agitation, altered consciousness, rigidity and convulsion. Although only a small portion of participants consumed energy drink ≥ 3 per a week and rarely exposed to caffeine for intoxicating levels, chronic caffeine overdose could result in adverse psychological and physical effects. Emotionally, caffeine overdose induces anxiety and irritability [1]. These bothersome psychological and physical symptoms might contribute to aggravating stress in adolescents with frequent energy drink intake.

The relation of energy drink intake with suicide might be partially influenced by the abnormal sleep time. Frequent consumption of energy drinks was associated with both long and short sleep times in this study. The sleep disturbance by caffeine intake was suggested for several studies but there has been few study showed U shape relation between sleep time and energy drink consumptions in adolescents [18, 23]. Stimulation of the adrenergic system could induce alertness and awakening, which would result in short sleep times, consistent with the results of the present study. In addition, caffeine reduces sleep quality by increasing wake time and decreasing deep sleep [10]. The consumption of energy drinks was found to be associated with approximately 1.50–1.88 times higher rates of poor sleep quality in college students [11, 24]. Especially, caffeine reduces the slow-wave or deep sleep in a dose-related manner and alters the sleep architecture of rapid eye movement (REM)/non-REM sleep [25, 26]. This low quality of sleep might result in daytime sleepiness and prolonged sleep time, as demonstrated in the present study [25].

The stress and poor school performance also could contribute to the association between high energy drink intake and suicide. The effects of high caffeine intake on stress and sleep could cause inefficient learning. Additionally, high caffeine intake has been related to depression and anxiety, which might reduce the discipline needed to study and induce disorientation from school work. Consequently, frequent energy drink consumption was proportionally related to lower school performance. Similarly, prior findings have shown a negative association between frequent energy drink intake and school performance [21, 27].

In addition to caffeine, additive compounds such as taurine, L-carnitine, glucuronolactone, B-vitamins, cocoa, kola nut, and guarana could contribute to the psychological symptoms related to energy drink consumption [6]. The types and amount of additive compounds in energy drinks vary. These additive compounds could interact with caffeine or act directly to generate psychological symptoms [6]. A cross-sectional study reported that energy drink users showed 3.12 times more behavioral or physiologic problems with trouble at home, school, or work than those who consumed beverages that were only caffeinated [28]. Furthermore, concomitant drug dependence may result in mental health problems [29]. Although there has been some controversy regarding this association, energy drinks have been suggested to be related with dependence on drugs such as alcohol [30]. Approximately 17.3% of adolescents have reported using energy drinks mixed with alcohol [30]. Compared to consuming alcoholic drinks alone, the consumption of energy drinks with alcohol has led to elevated stimulation, alertness, and the desire to continue drinking [31]. Frequent consumers of energy drinks in addition to alcoholic drinks were more likely to experience drug abuse [32]. Combining drug abuse with energy drinks might have detrimental mental and physical effects including on stress, sleep, and school performance and subsequently on suicide.

Although we considered several demographic and socioeconomic factors including age, sex, region of residence, economic level, fathers’ and mothers’ education levels, sleep time, stress level, and school performance, it is still possible that unadjusted socioeconomic factors affected the adverse mental health and behavioral outcomes observed in this study. Use of energy drinks is known to be related with socioeconomic factors [30]. For example, the desire to lose weight might be related to energy intake and stress. Males have been reported to drink more energy drinks than females, and gender was thus adjusted for in the present study; the findings indicated significant relations of energy drink consumption with stress, sleep, school performance, and suicide attempt, regardless of gender. However, causality could not be determined in the present study given the cross-sectional study design. High levels of stress, sleep deprivation, and low school performance could induce frequent energy drink consumption. A prospective cohort study should be conducted to determine the direction of the relationship. There are other limitations that should be mentioned. Stress level, sleep time, school performance, and energy drink intake were surveyed based on a self-reported questionnaire, which has inherent limitations regarding the validity of the data and the recall bias. Many beverages including tea, chocolate, cola, soft drinks and energy drinks contain caffeine at various concentrations. Therefore, the surveyed frequencies of energy drink use could not exactly represent caffeine consumption. However, many prior studies have also used the frequency of energy drink intake to represent high caffeine consumption, and the amount of energy drinks consumed was proportionally correlated with a plasma caffeine concentration of 1.2 to 25.4 mg/L in adolescents [33].

## Conclusion

Suicide attempts increased with frequent energy drink intake, independent of stress, sleep time, and school performance. Frequent consumption of energy drinks was also likely to be associated with more severe stress, inadequate sleep time, and lower school performance in Korean adolescents. This study suggests that the possible adverse effects of energy drink consumption in a population of highly-stressed students warrants further research.

## Supporting information

S1 Table Multinomial logistic regression analyses with complex sampling of subjective stress level, sleep time, and self-assessment of school performance for high energy drinking. (DOCX)

S2 Table Ordinal logistic regression analyses with complex sampling of subjective stress level, sleep time, and self-assessment of school performance for high energy drinking in each male and female group. (DOCX)

S3 Table Multiple logistic regression analyses with complex sampling of high energy drink for suicide trial in each male and female group. (DOCX)
