# Supplementary material for: High stress, lack of sleep, low school performance, and suicide attempts are associated with high energy drink intake in adolescents
Source: PLoS One. 2017 Nov 14;12(11):e0187759. doi: 10.1371/journal.pone.0187759 (PMC5685612; doi:10.1371/journal.pone.0187759)
Supplement: S1 Table — (DOCX) [file pone.0187759.s001.docx]

**S1 Table** Multinomial logistic regression analyses with complex sampling of subjective stress level, sleep time, and self-assessment of school performance for high energy drinking

|  | | High energy drink 1-2/week | High energy drink ≥ 3/week |  |
| --- | --- | --- | --- | --- |
|  | | AOR (95% CI) | AOR (95% CI) | P-value |
| Subjective stress level | |  |  | < 0.001* |
|  | No | 1 | 1 |  |
|  | A little | 1.03 (0.90-1.18) | 0.62 (0.51-0.75) |  |
|  | Mild | 1.27 (1.11-1.45) | 0.79 (0.66-0.94) |  |
|  | Moderate | 1.49 (1.30-1.69) | 1.08 (0.90-1.29) |  |
|  | Severe | 1.73 (1.50-1.99) | 2.17 (1.80-2.62) |  |
| Sleep time | |  |  | < 0.001* |
|  | < 6 h | 1.43 (1.34-1.52) | 2.08 (1.87-2.31) |  |
|  | ≥ 6 h, < 7 h | 1.09 (1.03-1.16) | 1.26 (1.03-1.16) |  |
|  | ≥ 7 h, < 8 h | 1 | 1 |  |
|  | ≥ 8 h, < 9 h | 0.99 (0.92-1.06) | 0.91 (0.80-1.04) |  |
|  | ≥ 9 h | 1.05 (0.96-1.14) | 1.43 (1.24-1.65) |  |
| Performance at School | |  |  | < 0.001* |
|  | A | 1 | 1 |  |
|  | B | 1.05 (0.97-1.13) | 0.93 (0.83-1.05) |  |
|  | C | 1.20 (1.11-1.29) | 1.04 (0.92-1.17) |  |
|  | D | 1.36 (1.26-1.47) | 1.19 (1.06-1.34) |  |
|  | E | 1.66 (1.52-1.82) | 1.53 (1.34-1.75) |  |

* Significance at P < 0.05
