# Supplementary material for: High stress, lack of sleep, low school performance, and suicide attempts are associated with high energy drink intake in adolescents
Source: PLoS One. 2017 Nov 14;12(11):e0187759. doi: 10.1371/journal.pone.0187759 (PMC5685612; doi:10.1371/journal.pone.0187759)
Supplement: S2 Table — (DOCX) [file pone.0187759.s002.docx]

**S2 Table** Ordinal logistic regression analyses with complex sampling of subjective stress level, sleep time, and self-assessment of school performance for high energy drinking in each male and female group

|  | | AOR (95% CI) | P-value |
| --- | --- | --- | --- |
| **Male** | |  |  |
| Subjective stress level | |  | < 0.001* |
|  | No | 1 |  |
|  | A little | 0.89 (0.79-1.02) |  |
|  | Mild | 1.10 (0.98-1.25) |  |
|  | Moderate | 1.33 (1.17-1.51) |  |
|  | Severe | 1.85 (1.60-2.14) |  |
| Sleep time | |  | < 0.001* |
|  | < 6 h | 1.60 (1.48-1.72) |  |
|  | ≥ 6 h, < 7 h | 1.15(1.07-1.24) |  |
|  | ≥ 7 h, < 8 h | 1 |  |
|  | ≥ 8 h, < 9 h | 0.97 (0.90-1.04) |  |
|  | ≥ 9 h | 1.16 (1.06-1.27) |  |
| Performance at School | |  | < 0.001* |
|  | A | 1 |  |
|  | B | 1.01 (0.92-1.10) |  |
|  | C | 1.13 (1.04-1.23) |  |
|  | D | 1.25 (1.15-1.37) |  |
|  | E | 1.52 (1.38-1.57) |  |
| **Female** | |  |  |
| Subjective stress level | |  | < 0.001* |
|  | No | 1 |  |
|  | A little | 0.84 (0.66-1.07) |  |
|  | Mild | 1.05 (0.83-1.33) |  |
|  | Moderate | 1.31 (1.03-1.65) |  |
|  | Severe | 1.88 (1.48-2.39) |  |
| Sleep time | |  | < 0.001* |
|  | < 6 h | 1.59 (1.46-1.72) |  |
|  | ≥ 6 h, < 7 h | 1.10 (1.01-1.20) |  |
|  | ≥ 7 h, < 8 h | 1 |  |
|  | ≥ 8 h, < 9 h | 1.00 (0.91-1.10) |  |
|  | ≥ 9 h | 1.13 (0.99-1.28) |  |
| Performance at School | |  | < 0.001* |
|  | A | 1 |  |
|  | B | 1.02 (0.91-1.14) |  |
|  | C | 1.18 (1.05-1.32) |  |
|  | D | 1.39 (1.24-1.55) |  |
|  | E | 1.78 (1.56-2.03) |  |

* Significance at P < 0.05
