# Supplementary material for: High stress, lack of sleep, low school performance, and suicide attempts are associated with high energy drink intake in adolescents
Source: PLoS One. 2017 Nov 14;12(11):e0187759. doi: 10.1371/journal.pone.0187759 (PMC5685612; doi:10.1371/journal.pone.0187759)
Supplement: S3 Table — (DOCX) [file pone.0187759.s003.docx]

**S3 Table** Multiple logistic regression analyses with complex sampling of high energy drink for suicide trial in each male and female group.

|  | | AOR (95% CI) | P-value |
| --- | --- | --- | --- |
| **Male** | |  |  |
| High energy drink | |  | < 0.001* |
|  | 0/week | 1 |  |
|  | 1-2/week | 1.47 (1.23-1.77) |  |
|  | ≥ 3/week | 3.35 (2.79-4.04) |  |
| **Female** | |  |  |
| High energy drink | |  | < 0.001* |
|  | 0/week | 1 |  |
|  | 1-2/week | 1.45 (1.24-1.70) |  |
|  | ≥ 3/week | 2.69 (2.17-3.35) |  |

* Significance at P < 0.05
